# Effects of various decellularization methods on histological and biomechanical properties of rabbit tendons

**DOI:** 10.3892/etm.2014.1742

**Published:** 2014-05-28

**Authors:** SHUXING XING, CONG LIU, BING XU, JIANCHANG CHEN, DONGFENG YIN, CHUNHAO ZHANG

**Affiliations:** Second Department of Orthopedics, Ürümqi General Hospital, Lanzhou Command, Ürümqi, Xinjiang 830000, P.R. China

**Keywords:** acellular, tendon, extracellular matrix, biomechanics, collagen

## Abstract

The aim of the present study was to investigate the effects of various decellularization methods on the histological and biomechanical properties of rabbit tendons. In total, six chemical reagents, including 1% t-octyl-phenoxypolyethoxyethanol (Triton-X 100), 0.5% sodium dodecyl sulfate (SDS), 1% tri-n-butyl phosphate (TnBP), 1% Triton-X 100 + 0.5% SDS, 1% TnBP + 0.5% SDS and 1% TnBP + 1% Triton-X 100, were used on rabbit semitendinosus muscles and flexor digitorum tendons for 24 h to remove cells. Hematoxylin and eosin staining was applied for histological observation, while tension testing was used for biomechanical studies. The effects of the various decellularization methods on the histological structure and biomechanical properties of rabbit tendons were evaluated. A group of fresh tendons treated with phosphate-buffered saline served as controls. The various decellularization methods resulted in different effects on the tendons. All the treatment groups exhibited a decrease in tendon biomechanical properties, but no statistically significant differences were observed among the experimental groups. The extensibility of the 1% TnBP-treated group was found to be greater than that of the other groups; however, the difference was not statistically significant. Histologically, the 1% TnBP + 0.5% SDS treatment was shown to have the least impact on the rabbit tendon structure, with good decellularization and no clear cellular remnants observed. The 1% Triton-X 100 + 0.5% SDS treatment had a pronounced effect on the tendon collagen structure and a number of collagen ruptures were observed. Overall, 1% TnBP + 0.5% SDS was found to be the most effective compared with the other treatments, as this treatment preserved the tendon collagen structure while completely removing the cells. Tendons treated with 1% TnBP + 0.5% SDS were histologically similar to normal tendon tissue and biomechanically similar to the tendons in the control group.

## Introduction

Anterior cruciate ligament (ACL) rupture is a common injury among young sportsmen and women, with a reported annual incidence in the general population of 0.8 per 1,000 (1). The ACL is a common site of knee joint injury. With the wide use of allogeneic tendons in cruciate ligament reconstruction, research into acellular tendons and acellular tissue-engineered tendons has become increasingly studied. Ideal tissue-engineered ligaments retain the basic histological structure of the ligaments and provide a suitable environment for stem cell proliferation and differentiation, forming connections between ligament collagen fibers. Acellular tissue-engineered ligaments provide a suitable alternative, while preventing ligament immunogenicity and the spread of disease (2). Therefore, further research is required to establish more effective and physiological graft options with a greater understanding of anatomical graft placement and graft tension (3). At present, tendon decellularization has been studied intensively with a variety of chemical reagents. The removal of ligament cell components may reduce immunogenicity, preserve the integrity of ligament structure and preserve the biological characteristics of the ligament. There are a number of decellularization methods, including physical, chemical and enzymatic, as well as combinations of these techniques (4). The ideal method is to remove cell residue completely, while maintaining collagen structure, in order to maximize the retention of the biomechanical characteristics of the tendon, leaving the original extracellular matrix (ECM) structure and composition intact. ECM materials may also encourage angiogenesis, which is required to deliver cells to repair the tissue and prevent infection (5). In previous years, tissue engineering has emerged as a promising approach for tendon repair or regeneration (6–9). The decelluarization tissue engineering tendon for ACL repair has beep reported as one of important method (10), however to find the most effective and least destructive decelluarization method was the important step.

Comparisons between singular and different combinations of decellularization methods have not yet been conducted, thus, in the present study, a variety of combinations were investigated, including t-octyl-phenoxypolyethoxyethanol (Triton-X 100), sodium dodecyl sulfate (SDS) and tri-n-butyl phosphate (TnBP), in order to determine which combination exhibited the greatest decellularization effect. In the present study, rabbit semitendinosus (ST) and flexor digitorum (DT) muscles were treated with a variety of decellularization methods and the histological and biomechanical changes were evaluated. Through comparing the effects of various methods on the collagen structure and biomechanics, the aim of the study was to identify an the most effective decellularization method.

## Materials and methods

### Animal grouping

In total, 28 male standard New Zealand rabbits (the experimental animals were provided and maitanied by the Experimental Animal Center of Urumqi Gengeral Hospital), weighing 3.0–3.5 kg, were randomly divided into control and experimental groups. A total of eight ST and DT sections from the hindlimb flexor muscles were harvested from each group and immediately prepared for testing. Four rabbits comprised the control group, and the experimental group was further divided into six subgroups of four animals each. A total of 48 ST and DT samples were collected and the tendons were treated with six decellularization methods. The study was conducted in strict accordance with the recommendations in the Guide for the Care and Use of Laboratory Animals of the National Institutes of Health, and the animal use protocol was reviewed and approved by the Institutional Animal Care and Use Committee of Ürümqi General Hospital (Ürümqi, China).

### Tissue extraction and storage

Tendons were collected from the rabbits following the administration of anesthesia. All the tendons and accessory tissues were removed and stored at 4°C in phosphate-buffered saline (PBS) with 5% penicillin/streptomycin (Mediatech, Herndon, VA, USA) and 0.02% EDTA (Sigma-Aldrich, St. Louis, MO, USA) to prevent tissue degradation and inhibit bacterial growth. All the specimens were washed at 4°C with PBS for 12 h prior to the experiments.

### Decellularization methods

Tendons were treated with the following six chemical reagents for 24 h: i) 1% Triton-X 100 (Yuanye Biotechnology Co., Shanghai, China); ii) 0.5% SDS (Yuanye Biotechnology Co.); iii) 1% TnBP (Yuanye Biotechnology Co.); iv) 1% Triton-X 100 + 0.5% SDS; v) 1% TnBP + 0.5% SDS; and vi) 1% TnBP + 1% Triton-X 100. The control group was treated with PBS (Cellchip Biotechnology Co., Beijing, China) only.

Following the decellularization treatment, the tendons were washed in distilled water at 4°C for 24 h. Next, the tendons were incubated in PBS containing 100 μg/ml DNase (Sigma-Aldrich) and 150 IU/ml RNase (Sigma-Aldrich) at 37°C for 24 h, and then placed in PBS containing 0.02% EDTA at 4°C for 24 h. All the specimens were stored in a gauze at 4°C with PBS until required for mechanical testing and pathological analysis.

### Histopathological analysis

Gross observations analyzing the tendon morphology and color were conducted. A number of the tendons from the various groups were selected to assess the extent of decellularization and collagen changes using light microscopy (Olympus Corporation, Tokyo, Japan).

### Measurements

Cross-sectional dimensions were measured using vernier calipers. The tendons were extended straight with no tension and the cross-sectional area was calculated, assuming an elliptical cross-section. The two ends of the tendon were placed at the two fixtures of the MTS 858 Mini Bionix test system (MTS Systems Corporation, Eden Prairie, MN, USA) and maintained at an upright position, straightening without tension by adjusting the test system. The distance between the two fixtures was measured and this was considered to be the length of the tested tendons. For rupture testing, a load was applied directly to tendons at constant velocity of 10 mm/min until the tendon broke. The test system recorded the load-displacement curve, the peak load of the tendon damage and the tensile displacement at the peak load. The rigidity and elastic modulus of the tendon graft were then calculated.

### Statistical analysis

Data were analyzed using SPSS 12.0 software (SPSS, Inc., Chicago, IL, USA) and the results are presented as the mean ± standard deviation. Biomechanical differences among the treatment groups and between the treatment and control groups were analyzed using the t-test, where P<0.05 was considered to indicate a statistically significant difference.

## Results

### Gross anatomy and pathology observations

**Figure 1:**
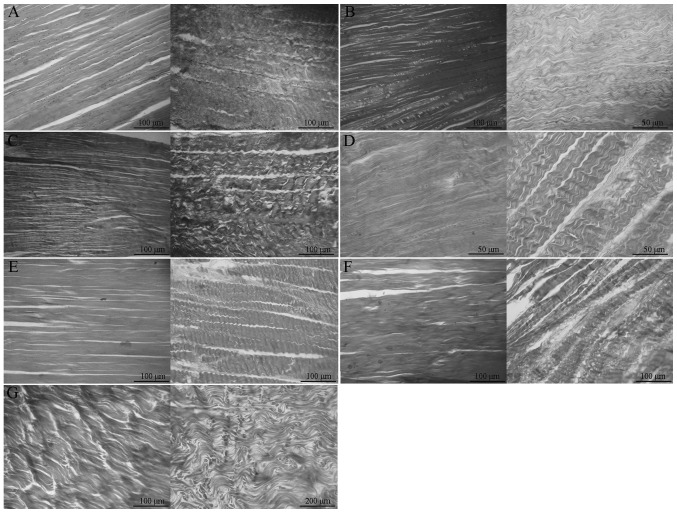
Rabbit limb flexor DT muscle (left, DT; right, ST) arrangement following treatment with (A) PBS (control), (B) 1% Triton-X 100, (C) 1% SDS, (D) 1% TnBP, (E) 1% TnBP + 0.5% SDS, (F) 1% TnBP + 1% Triton-X 100 and (G) 1% Triton-X 100 + 0.5% SDS decellularization methods. ST, semitendinosus ; DT, digitorum; Triton-X 100, t-octyl-phenoxypolyethoxyethanol; SDS, sodium dodecyl sulfate; TnBP, tri-n-butyl phosphate; PBS, phosphate-buffered saline.

In the group of untreated rabbit tendon specimens, the hematoxylin and eosin staining was normal, with tendon cells and normally arranged collagen structures observed, as shown in Fig. 1A. In the 1% Triton-X 100-treated group, as shown in Fig. 1B, a number of residual tendon cells were observed in the rabbit flexor DT muscles, while a small number of cells were observed in the ST muscles. In addition, the collagen structure was abnormal in the ST samples. In the 0.5% SDS-treated group, as shown in Fig. 1C, there was a small number of residual tendon cells, and the collagen arrangement and nuclear structures in the flexor DT muscles were normal. A small number of tendon cells and a small amount of vascular epithelial cell debris were observed in the ST muscles, with the arrangement of collagen largely normal with the exception of a few local ruptures. In the 1% TnBP-treated group, as shown in Fig. 1D, a small number of residual cells was observed in the flexor DT muscles, while in the ST samples, there were no cells remaining and the collagen gap had widened, revealing a small number of ruptures. In the 1% TnBP + 0.5% SDS-treated group, as shown in Fig. 1E, no cells were observed in the flexor DT or ST muscles. Collagen structures remained intact with a small number of ruptures and the collagen gap had widened slightly. In the 1% TnBP + 1% Triton-X 100-treated group, as shown in Fig. 1F, the treatment was effective. There were no clear cell remnants in the flexor DT or ST muscles, and the collagen arrangement was slightly disordered, particularly in the ST samples. In the 1% Triton-X 100 + 0.5% SDS-treated group, as shown in Fig. 1G, there were no clear residual cells in the flexor DT or ST muscles, however, the two areas exhibited disorganized collagen and evident collagen ruptures. Following the decellularization treatment, the two types of tendon exhibited a degree of damage to the collagen structures, particularly in the 1% Triton-X 100- and 1% Triton-X 100 + 0.5% SDS-treated groups. The three groups treated with a single reagent all showed residual cells, particularly in the DT muscle, which is bigger and thicker compared with the ST muscle. In the groups treated with two reagents, the treatment was more effective and there were no residual cells observed in the tendons. In the 1% TnBP-treated group, little damage to the collagen structure was observed. In the 1% TnBP + 0.5% SDS-treated group, the collagen structure remained intact and only a small amount of collagen fracturing was observed in the ST muscles. In the 1% Triton-X 100 + 0.5% SDS-treated group, the treatment was effective, however, significant collagen damage was observed. Therefore, the 1% TnBP + 0.5% SDS treatment preserved the integrity of the tendon collagen arrangement and structure, while successfully removing all the nuclei.

### Tensile biomechanics

**Figure 2:**
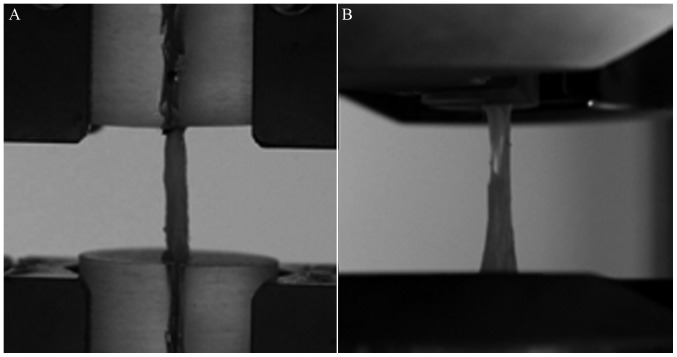
Images showing a tendon at (A) mechanical testing and (B) rupture at maximum load.

Tendons were subjected to tensile mechanical testing (Fig. 2). All the specimens failed at the ligament section. No damage was observed at the tendon or clip surface, however, tendon ruptures were located at the body of the tendon, close to the flat end of the tendon.

**Figure 3:**
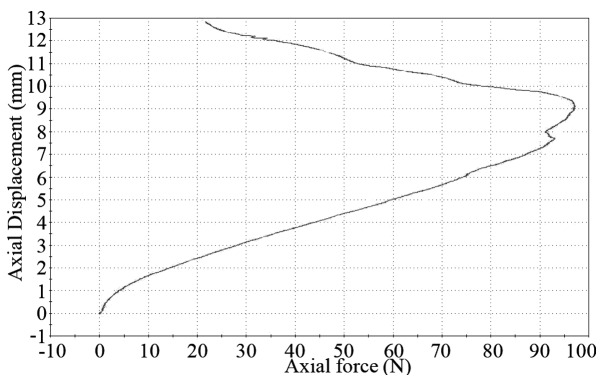
Stress-strain curve of a typical tendon mechanical testing.

A typical stress-strain curve at the tendon stretch is shown in Fig. 3. The association between the stress and strain that a particular material displays is known as that particular material’s stress-strain curve. It is unique for each material and is calculated by recording the amount of deformation (strain) at distinct intervals of tensile or compressive loading (stress). It shows certain properties of the tendon.

No statistically significant difference was detected in the morphological parameters between the ST and flexor DT muscles (Table I).

Final mechanical measurements are shown in Table II. No statistically significant differences were observed among the three types of structural failure loads (normal ST, 101.40±6.34 N; normal flexor DT, 253.78±12.36 N). The 1% Triton-X 100 + 0.5% SDS group exhibited a lower failure load, however, no statistically significant difference was detected among the groups. With regard to extensibility, statistically significant differences were observed between the TnBP and control groups, as well as between the TnBP and other treatment groups. TnBP treatment was found to significantly increase the extensibility. No statistically significant difference was observed in extensibility among the other groups. The results indicated that the extensibility of the tendons increased following TnBP treatment, and the increase was statistically significant when compared with the other groups. Therefore, TnBP treatment may cause the relaxation of tendons. No statistically significant difference with regard to failure load or elastic modulus was observed between the TnBP treatment group and the other groups. The elastic modulus of the 1% Triton-X 100 + 0.5% SDS-treated group was significantly different when compared with the control group (P<0.05), however, statistically significant differences were not observed when compared with the other treatment groups (P>0.05).

## Discussion

The present study analyzed the decellularization effects of two rabbit tendons treated with several singular and combination decellularization methods, with the aim of determining the most effective decellularization method. These observations may provide rational for the future study of tendon tissue engineering applications. At present, decellularization methods focus on a number of chemical reagents. These decellularization methods are frequently used in research, however, the most effective method of decellularization for the rabbit tendon has not yet been reported in literature. Thus, the present study compared the effects of a variety of decellularization methods on the histological structures and biomechanical properties of rabbit flexor DT and ST muscles.

Decellularizated tendons, as compared with allografts or synthetic materials, have a number of promising advantages. Firstly, the immunogenicity and antigenicity of the tissue is reduced, whereas only minimal antigenic ECM is preserved (11). Secondly, an ideal environment is provided for the cells to undergo incorporation, metabolism and matrix synthesis, by preserving other macromolecules in addition to the collagenous structure. Furthermore, by preserving the natural structure of the tendon, the initial biomechanical strength should be preserved, with a biomechanical strength similar to that of the tendon itself (12).

The results of the present study demonstrated that all the decellularization methods have the capacity to remove cells from the tendon. Histological results showed intact cells and cell debris following decellularization with a single reagent. Fewer cells remained in the ST muscle, as compared with the flexor DT muscle, which may be due to the smaller diameter of the ST. Combinations of two reagents exhibited excellent decellularization effects and there were fewer intact cells and cell debris when compared with the single reagent methods. The 1% TnBP + 1% Triton-X 100 and 1% TnBP + 0.5% SDS treatments exhibited excellent decellularization effects with a predominantly intact collagen structure. In particular, the 1% TnBP + 0.5% SDS treatment demonstrated little damage to the collagen structure, with the collagen showing a normal arrangement. Tendons treated with 1% Triton-X 100 + 0.5% SDS exhibited more ruptures in the collagen structure and more disordered arrangements when compared with the other treatment groups. Biomechanical analysis indicated that all the decellularization treatments maintained a good biomechanical performance. The 1% TnBP + 0.5% SDS group exhibited the least mechanical reduction, while the 1% Triton-X 100 + 0.5% SDS group demonstrated the most biomechanical reduction. This observation may be due to the intensive collagen damage associated with this method. No statistically significant difference was observed with regard to biomechanics between each treatment group and the control. The results also demonstrated that the extensibility of the ST and DT muscles increased following TnBP treatment, with the difference between the control and TnBP treatment group being statistically significant (P<0.05). No statistically significant difference in extensibility was observed between the other treatment groups and the control group. The study conducted by Deeken *et al*, which used TnBP (1%) to treat pig diaphragmatic tendon specimens, demonstrated TnBP to have excellent decellularization effects, but also increased tendon extensibility (13). The increased extensibility associated with TnBP treatment may be due to the effects of TnBP on collagen crosslinking. TnBP treatment also makes it easier for collagen fibers to slide and cause the entire tissue to extend easily, which may also be due to the decreased degree of crosslinking. The increased tendon extensibility associated with TnBP treatment may lead to tendon laxity following reconstruction, which may affect the long-term biomechanical properties of the tendon. Therefore, TnBP treatment is not an ideal decellularization method and the present study does not recommend TnBP treatment for the preparation of tendon scaffolds. However, TnBP treatment may be combined with other methods in order to mitigate the effects on tendon extensibility.

Tendon damage and cruciate ligament injury are the main causes of lost and limited motor functions (14,15). Ligaments have a very limited ability to self-heal following injury and often require surgical intervention. This is particularly true of cruciate ligament injury following knee joint torsion. For young patients, cruciate ligament reconstruction using autologous tendons is an ideal method to restore function, however, this method may cause donor site complications, including sustained patellar pain, a lack of muscle strength around the knee joints and a decline in joint stability. Allografts are an alternative material for surgical reconstruction, however, these may cause immune responses and spread disease. The long-term results associated with allogeneic tendons are not good. For example, they involve risks of ligament laxity and extension and decreased mechanical properties. Acellular tissue-engineered ligaments are becoming increasingly studied (16) and research has shown that tissue-engineered ligaments are the most promising method of solving this problem. The success of tissue engineering relies on the selection of optimal biological materials, and acellular ligaments may be the most effective. Decellularization technology includes physical methods, such as ultrasound, freezing and agitation; chemical methods, such as organ-dissolving solutions and acid, ionic, nonionic and zwitterionic detergents; and enzymes, such as proteases, nucleases and combinations thereof. Ideal decellularization techniques successfully dissolve the cell membrane, cytoplasm and nuclear remnants, while removing the residual chemical and cellular components, leaving the original ECM scaffolds and their optimal biomechanical properties undamaged (17). The results of previous studies have led researchers to question whether the acellular tendon is necessary for ligament tissue engineering. The removal of zymogen clusters from cell surfaces using antigenic determinants can reduce allograft immunogenicity (18). Inner cells can cause necrosis following transplantation and delay host cell invasion and religamentization. Decellularization may reduce the allogeneic spread of disease. This evidence cannot be ignored (19). Therefore, the decellularization of tendon scaffolds continues to be necessary for ligament tissue engineering (20). Decellularization using SDS, an ionic detergent, Triton-X 100, a nonionic detergent, and TnBP, a disinfecting and bacteriostatic agent, has been successfully applied in scaffold preparation for skin grafts, heart valves, blood vessels, tendons, ligaments and cartilage (21,22).

Studies conducted by Cartmell and Dunn on patellar tendon grafts demonstrated that TnBP and SDS decellularization treatment removed 70–90% of cells (23,24). Only a few cells remained, although a number of changes to the collagen morphology were observed. Similar results were obtained when the authors used SDS to treat rat tail tendons. Specimens treated with Triton-X 100 exhibited numerous broken cells, cell debris and mild collagen damage. However, a study conducted by Courtman *et al* demonstrated that Triton-X 100 successfully removed cells in other tissues, including bovine pericardium, without damaging the collagen structure or reducing the collagen strength (25). In the present study, the results showed that all the decellularization methods exhibited a certain capacity to remove cells, with the methods involving a single reagent showing better retention of the collagen structure. Among the methods involving two reagents, the 1% TnBP + 0.5% SDS treatment exhibited the least collagen damage.

The study conducted by Woods and Gratzer focused on the effects of Triton-X combined with SDS (26). The results demonstrated that this combination had excellent decellularization effects, however, it also clearly damaged the glycosaminoglycan and increased the collagen tensile strength. The current study also demonstrated that the combination of Triton-X 100 and SDS caused collagen disorganization, local rupture and decreased biomechanical strength, but this decrease was not significant when compared with the other treatment groups. Histological examinations performed in the current study revealed that Triton-X with SDS effectively removed cells from the ST muscles, but not from the robust flexor DT muscles, which had cells remaining. This observation indicated that more time was required to completely remove the cells from the flexor DT muscles, and that the removal of these cells may increase the collagen damage. Thus, joint application of different decellularization reagents is recommended. For example, the present study found that the combination of TnBP and SDS completely removed cells from the rabbit ST and flexor DT muscles, while leaving the collagen structure undamaged. A minimal decrease in biomechanical strength was observed and no statistically significant difference was identified when compared with the normal tendon tissue. When two reagents are used in combination, they compliment each other and produce improved decellularization effects, while leaving the tendon integrity and biomechanical strength undamaged.

## Figures and Tables

**Table I:** Comparison of cross-sectional areas and lengths of the tendons in the various decellularization groups (mean ± SD).

	Cross-sectional area (mm^2^)		Length (mm)	
		
Group	ST	DT	ST	DT
1	1.02±0.15	3.02±0.68	20.65±1.23	20.20±0.84
2	0.85±0.18	2.12±2.34	18.67±0.14	20.91±1.13
3	1.22±0.75	2.42±1.25	20.07±0.63	18.87±1.89
4	0.94±0.67	2.48±1.70	20.24±1.08	18.43±1.68
5	0.73±0.36	2.53±1.94	19.98±0.35	19.56±1.05
6	0.89±1.04	2.47±0.88	20.16±0.76	20.00±0.81
7	0.92±1.31	2.82±1.53	19.38±1.28	20.12±1.32

Groups: 1, 1% Triton-X 100; 2, 0.5% SDS; 3, 1% TnBP; 4, 1% Triton-X 100 + 0.5% SDS; 5, 1% TnBP + 0.5% SDS; 6, 1% TnBP + 1% Triton-X 100; 7, control (PBS-treated). ST, semitendinosus; DT, digitorum; PBS, phosphate-buffered saline; Triton-X 100, t-octyl-phenoxypolyethoxyethanol; SDS, sodium dodecyl sulfate; TnBP, tri-n-butyl phosphate.

**Table II:** Failure load, extensibility and elastic modulus of the tendons in the various decellularization groups (mean ± SD).

	Failure load (N)		Elastic modulus (MPa)		Extensibility (%)	
			
Group	ST	DT	ST	DT	ST	DT
1	88.63±8.70	225.80±16.05	317.25±2.03	492.76±3.47	11.98±4.24	9.27±2.83
2	92.02±7.76	233.4±23.92	331.43±5.14	503.76±5.86	12.71±1.52	10.63±1.42
3	85.74±10.13	234.58±28.31	285.67±11.73	483.76±2.64	25.52±8.23^a^	22.78±4.18^a^
4	81.36±7.63	198.24±21.05	313.52±7.76^b^	493.76±7.55^b^	13.56±3.03	10.36±3.86
5	93.23±7.04	238.77±27.13	332.76±2.19	538.76±4.62	13.52±2.16	12.76±0.93
6	90.82±7.85	235.82±12.59	330.34±5.33	529.76±6.32	14.75±1.89	11.66±2.53
7	101.40±6.34	253.78±12.36	356.52±16.94	560.09±2.73	10.73±3.21	8.76±2.76

Groups: 1, 1% Triton-X 100; 2, 0.5% SDS; 3, 1% TnBP; 4, 1% Triton-X 100 + 0.5% SDS; 5, 1% TnBP + 0.5% SDS; 6, 1% TnBP + 1% Triton-X 100; 7, control (PBS-treated). No significant difference was observed in the failure load among the groups or between any experimental group and the control group (P>0.05).

a For extensibility, the TnBP group was significantly different when compared with the other groups and the control group (P<0.05).

b For elastic modulus, the 1% Triton-X 100 + 0.5% SDS group was significantly different from the control group (P<0.05), but not significantly different when compared with the other groups (P>0.05).

ST, semitendinosus; DT, digitorum; Triton-X 100, t-octyl-phenoxypolyethoxyethanol; SDS, sodium dodecyl sulfate; TnBP, tri-n-butyl phosphate; PBS, phosphate-buffered saline.
